# Insights into middle-aged men (40–70) who died by suicide: a nationwide mixed methods study in the Netherlands

**DOI:** 10.1186/s12889-026-27854-8

**Published:** 2026-06-02

**Authors:** Jonas Manait, Yvonne Luigjes-Huizer, Guus Berkelmans, Anne C Miers, Elias Balt, Stefan Vrinzen, Irene van Herrewegen, Saskia Mérelle, Laura Shields-Zeeman, Renske Gilissen

**Affiliations:** 1 Research department, 113 Suicide Prevention, Amsterdam, the Netherlands; 2 Department of Clinical Psychology, Leiden University, Leiden, the Netherlands; 3 Department of Mental Health and Prevention, Trimbos Institute, Utrecht, the Netherlands; 4 Department of Developmental and Educational Psychology, Leiden University, Leiden, the Netherlands; 5 Department of Interdisciplinary Social Science, Utrecht University, Utrecht, the Netherlands; 6 Amsterdam Public Health School, Amsterdam University Medical Center, Amsterdam, the Netherlands; 7 Department of child and adolescent psychiatry and psychosocial care, Amsterdam University Medical Center, Amsterdam, the Netherlands

**Keywords:** Suicide, Middle-aged men, Risk, Population data, Machine learning, Psychosocial autopsy

## Abstract

**Background:**

Globally, middle-aged men dominate suicide statistics in many regions. To understand their heightened vulnerability, we examine the interplay between sociodemographic factors, contextualized through psychosocial perspectives.

**Methods:**

Data from Statistics Netherlands of all middle-aged men (40–70) who died by suicide between 2012 and 2021 (*N* = 6,656) and interview data from a psychosocial autopsy study of suicide decedents (*n* = 25) were combined. We applied a machine learning method on the population data to detect subgroups of middle-aged men with a higher risk of dying by suicide. To distil factors and nuances beyond sociodemographic categorization, we executed a thematic analysis on the interview data from the psychosocial autopsy study.

**Results:**

On average the suicide rate for middle-aged men in the Netherlands was 21.3 per 100,000. The suicide rate was high (> 40 suicides per 100,000) for middle-aged men who received mental health care (114), received a disability benefit (55.9), lived alone (51.6), received social assistance (50.6), had somatic health care costs over €10,000 per year (49), or between €5,000 and €10,000 per year (43), or had a low household income (40.8). However, it became evident that suicide rates were significantly higher when these factors accumulated, with the combination of (i) received mental health care, (ii) lived alone, and (iii) received a disability benefit resulting in 289.1 suicides per 100,000. The thematic analysis contextualized sociodemographic risk factors by providing insight into underlying themes, including the experience of loss, disconnectedness, and debilitation.

**Conclusions:**

The data indicate that the accumulation of (mental) health, socioeconomic, and interpersonal hardships, carry significant risk for middle-aged men. The interaction of factors underscores the complexity of the origin of suicide, while also pointing to avenues for prevention efforts. Suicide prevention efforts must be integrated into the systems and contexts where middle-aged men are currently situated, which include clinical care, community settings, and social services. Future research should explore the support needs of middle-aged men with lived experience.

**Supplementary Information:**

The online version contains supplementary material available at 10.1186/s12889-026-27854-8.

## Background

Suicide represents a major cause of premature death worldwide [1], with every suicide estimated to affect up to 135 people [2]. In many regions globally, men have a higher risk of dying by suicide than women [1, 3]. High-income countries show the most pronounced male predominance in suicide, with the highest rates observed among middle-aged men [4]. This is well illustrated in the Netherlands, where middle-aged men (aged 40–70) have been dominating suicide statistics for decades [5].

Midlife is known as a period marked by unique challenges and distress [6]. This stems from shifts in overall health (e.g., physical decline), social relations (e.g., changes in family composition and dynamics), and economic landscape (e.g., labor market volatility [7]). Over time, the accumulation and potential burden of these changes can intensify distress among men, heightening their vulnerability to suicidal ideation [8]. Sociodemographic and psychosocial factors such as having a low income, limited education, being unemployed, and having a mental illness diagnosis are associated with suicide among middle-aged men [9, 10]. Other contributing factors include relationship status (being unmarried, single or divorced [11]), physical ill-health, alcohol and/or drug misuse, bereavement [12], and exposure to childhood adversity [13, 14]. The complexity of understanding these contributing factors lies not in their individual impact, but rather in their dynamic interplay and cumulative effects [15]. Hence, more research is needed of how factors interact to impact suicide among middle-aged men.

Current theoretical models of suicide risk factors, such as proposed by Pirkis [16], acknowledge how systems of individual factors and social determinants interact and influence suicidality. Even when limiting attention to individual factors, there seems to be a high level of complexity where distal (indirect) and proximal (direct) factors affect one another [17]. Besides the contribution of Graney [12] in examining risk profiles among middle-aged (40–55) men, the current body of evidence remains limited. Many studies focus on single factors, include all adults or do not distinguish between genders [9, 10].

Understanding the interaction of multiple factors requires both large-scale datasets and insights from personal level experiences. Machine learning-based quantitative methods are increasingly applied on large-scale datasets to identify suicide risk factors [18–20] and uncover cumulative effects of sociodemographic variables that define significant risks [21]. However, these studies provide limited insight into underlying dynamics [20]. Methods that do consider these mechanisms include qualitative psychosocial autopsy studies, where proxy informants provide detailed information surrounding the psychosocial circumstances of an individual’s death [22–24]. Combining both machine learning and psychosocial autopsy methods into one study is therefore expected to offer a more comprehensive understanding of suicide risk, with the potential to generate richer insights than either method alone. Moreover, it brings together multiple perspectives in a manner that, to our knowledge, has not been attempted before.

The present mixed-methods study applies a machine learning method to examine the interplay of sociodemographic factors among middle-aged men who died by suicide in the Netherlands between 2012 and 2021, characterizing novel subgroups that have an increased risk of dying by suicide. Additionally, this study draws insights from psychosocial autopsy interviews to gain a deeper understanding of how different factors are perceived to contribute to suicide, thereby contextualizing and extending patterns that population data alone do not capture. The primary objectives are to identify risk patterns and to provide contextualization of the underlying dynamics, which is crucial to the development of targeted suicide prevention interventions for middle-aged men [9, 15, 17, 25]. In line with this objective, this study employs a hypothesis-free, data-driven approach, allowing for the identification of previously unidentified subgroups of middle-aged men that died by suicide.

## Methods

This mixed methods study design uses non-public nationwide microdata (i.e., individual level anonymized records) of Central Bureau of Statistics Netherlands (CBS) combined with interview data from a psychosocial autopsy study (post-mortem interviews). Combining macro- and micro-level data of middle-aged men who died by suicide allows for profile recognition while capturing psychosocial circumstances that underlie them. The Medical Research Ethics Committee of Amsterdam University Medical Center approved the psychosocial autopsy study (2023.0246).

### Nationwide statistics data

#### Data selection and study procedures

Microdata from Statistics Netherlands was used to analyze the sociodemographic characteristics of middle-aged men (40–70). Statistics Netherlands provides project-specific access via secure servers, with all results checked for privacy compliance. Data of men who were registered in the Netherlands between 01/01/2012 and 31/12/2021 was analyzed for this study, as data beyond 2021 were incomplete at the time of analyses. From 2012 to 2020, observations on age, mortality and cause of death (ICD10) were made on December 31 of each year for every individual. Men aged 40 to 70 were selected from these observations. Next, the following dependent variable was formulated: “Death by suicide in the calendar year following observation” based on ICD10 codes in the range X60-X84. All 6,656 men who died by suicide were included. Independent variables were guided by previous national data suicide research among Dutch inhabitants by Berkelmans [21, 26], and included: age, migration background, personal income, household income, financial household assets, physical problems measured as healthcare costs, mental health problems according to mental healthcare costs, household status, benefits, marital status, education level, and residential region.

#### Variables

Age was grouped in ten-year categories (40–49, 50–59, and 60–69). Residential region was divided into the North, East, South and West of the Netherlands. Migration background was divided into men without a migration background, first generation (non-)Western and second generation (non-)Western migration background [27]. Education level was categorized following the Dutch guidelines into lower (primary school, secondary school, first three years of college preparatory education [International Standard Classification of Education (ISCED) 1–2]), middle (last two to three years of college preparatory education [ISCED 3–4]), higher (college or university [ISCED 5–8]) and unknown. Marital status categories included: never married or in a registered partnership, married or in a registered partnership, widowed, divorced. Household status consisted of four categories: living with a child, living alone, living with a partner and with child(ren), and living with a partner without child(ren) living at home. Personal income, household income and household wealth were each categorized into four quartiles, with the fourth quartile representing the highest and the first quartile representing the lowest levels of income or wealth. Employment was coded dichotomously. Benefits were categorized as the presence or absence of unemployment benefits (i.e., Unemployment Act), incapacity or disability benefits (i.e., Incapacity benefit for self-employed persons, Incapacity Benefits Act, Work and Income according to Labor Capacity Act), and social assistance (i.e., National Assistance Act, Working and Social Assistance Act). Healthcare costs per calendar year (without mental health care) was categorized as: less than €5000, between €5000 and €10,000, more than €10,000 or unknown. Having mental health problems was classified as receiving mental health care (yes/no) per calendar year.

#### Analyses

For each variable, the number of suicides per 100,000 middle-aged men was calculated and compared with the total number of suicides per 100,000 for middle-aged men. A machine learning extension of logistic regression according to the method of Berkelmans [21] was used to identify factors associated with suicide, individually as well as in interaction. To prevent overfitting, the dataset used to select interaction terms is entirely separate from the dataset used to fit the final logistic regression model. This separation ensures that the final model remains interpretable: the selected interactions serve as pre-specified hypotheses, comparable to those a domain expert might propose based on prior knowledge, rather than interactions derived from the same data used for model estimation. Odds ratios (ORs) were derived from logistic regression to quantify suicide risk associated with individual predictors, while compound odds ratios (CORs; odds ratios for the combined effect of all factors in the interaction being present compared to none being present) were calculated to assess cumulative risk. Combined interactions were filtered on combinations that substantially improved model fit (log-likelihood), showed statistical significance after correction for number of tests, and involved groups with sufficient case numbers to correct for false positives. Because the factor receiving mental health care was dominant, we also present combinations without this variable, aiming to increase the relevance for men not receiving such care. We present the combinations with 30 or more suicides per 100,000. The dataset was split as follows: 50% of the cases that resulted in suicide along with 1% of the cases that did not result in suicide (control group) in the training set. The validation set consisted of 40% of cases that resulted in suicide and 1% random sample of the control cases. The final size of the validation set was 314,676 cases (person-years), of which 2,675 were suicides. To evaluate potential overfitting, a separate test set containing 10% of suicide cases and 1% sample of a control group was constructed, on which model performance was assessed. Analyses were conducted with the Python script used in Berkelmans [21].

### Interview data

#### Data selection and study procedures

Qualitative data from an ongoing psychosocial autopsy study in the Netherlands was used, involving proxy informants of middle-aged men who died by suicide. Recruitment procedures are described in detail in Balt [22]. In this study, participants were excluded if they were younger than 18, were admitted to a care facility at the time of the study irrespective of psychopathology, received advice against participation in the study from a general practitioner, or lacked Dutch language proficiency. Interviews were conducted face-to-face in the home of respondents by trained (retired) practitioners and psychologists experienced in psychosocial autopsy studies. No relationship with the participants was established prior to the study, and interviews were not repeated. Interviews lasted on average one-and-a-half hours and took place in 2023 and 2024, involving suicides that occurred between 2020 and 2024. A total of 30 interviews were included with proxy-informants of 25 middle-aged men who died by suicide; these comprised all processed interviews available, without further selection. The middle-aged men who died by suicide had a mean age of 54.7 (SD 7.7) and mainly had no migration background (92%); a detailed group description can be found in Additional file 1. For five of the deceased, two proxy-informants were available and included, to obtain more accurate and comprehensive data [28]. The primary informants were partners, ex-partners, parents, daughters, brothers and other family members. Secondary informants were identified via the primary informants and included friends and children. Prior to the interviews, a questionnaire obtaining standard information and empirically established risk factors about the deceased was completed by the proxy-informants. This information was used to describe the research population (e.g., mental health disorders, physical state, medicine use, work status).

#### Interview instrument

The interview instrument was developed based on previous international autopsy studies [29, 30] [see Additional file 2]. It involved three main sections, with the first one being a narrative account of the bereaved about factors contributing to suicide. The second part focused on a reconstruction of the last months of the deceased’s life, capturing the location and circumstances of the suicide, triggering factors, and adverse life events. The final section focused on addressing psychosocial risk factors. More details are described in the study by Balt [22].

#### Analyses

Audio files were transcribed verbatim. This analysis builds on previous work that identified unique psychosocial risk factors among middle-aged men (e.g., financial strain and adverse life events) [22]. Fine-grained secondary analyses were applied to describe themes that helped elucidate the factors identified in the nationwide statistics. Furthermore, the analyses aimed to identify factors that previously remained unexplored. The Thematic Analysis of Braun & Clarke [31] was utilized to this end. This method is widely used for Psychosocial Autopsy Data [32, 33] and involves six–recursive-phases of: familiarization with the data; generating initial codes; generating initial themes; reviewing and developing themes; refining and naming themes; and producing the final analysis. A deductive code list was employed that had previously been developed and applied to a larger dataset of the psychological autopsy in the Netherlands [22]. The list included a wide range of psychosocial factors (e.g., work-related problems, financial issues, burdensomeness, and bullying). Also, inductive coding was performed to identify codes that align with the research aim. JM and IvH coded the interviews. To avoid bias, both researchers worked independently before comparing their interpretations. The data analysis software *Atlas.ti* was used for analyzing the transcripts. The consolidated criteria for reporting qualitative research (COREQ) have been followed [34] [see Additional file 3].

## Results

### Population statistics

#### Individual factors

The average suicide rate for middle-aged men in the Netherlands was 21.25 per 100,000. Table 1 presents the results of the logistic regression model including the number of suicides, suicide rates, and the relative odds ratios across different sociodemographic categories. The suicide rate was notably higher (> 40 suicides per 100,000) for middle-aged men who received mental health care (114 suicides per 100,000), received a disability benefit (55.92 suicides per 100,000), lived alone (51.61 suicides per 100,000), received a social assistance benefit (50.65 suicides per 100,000), had somatic health care costs over €10,000 per year (49.62 suicides per 100,000), or between €5,000 and €10,000 per year (43.05 suicides per 100,000), or had a low household income (40.79 suicides per 100,000). There was also an increased number of suicides (30–40 suicides per 100,000) for middle-aged men who had a low income, received unemployment benefits or who were widowed, unmarried or divorced.

**Table 1:** Individual factors outcomes logistic regression model middle-aged (40–70) men from 2013 to 2021

Factors	Subcategories	Number of suicides (%)	Number of suicides per 100,000 middle-aged (40-70) men	Odds Ratio (CI)
Socio-demographic				
Age	Age 40–49 (Ref)	914 (34)	20.74	1.00 (1.00–1.00)
	Age 50–59	1084 (41)	24.45	1.15 (1.03–1.30)
	Age 60–69	677 (25)	18.07	0.72 (0.62–0.83)
Living region	Northern (Ref)	309 (12)	25.19	1.00 (1.00–1.00)
	Southern	719 (27)	23.22	1.00 (0.87–1.16)
	Western	1044 (39)	18.96	0.83 (0.72–0.95)
	Eastern	588 (22)	21.91	1.00 (0.87–1.14)
Migration background	No migration background (Ref)	2295 (86)	22.50	1.00 (1.00–1.00)
	1st Gen. Western Migration Background	114 (4)	21.34	0.82 (0.66–1.01)
	2nd Gen. Western Migration Background	144 (5)	21.75	0.88 (0.74–1.06)
	1st gen. non-Western^b^	107 (4)	9.63	0.30 (0.24–0.38)
	2nd gen non-Western	15 (1)	18.47	0.64 (0.38–1.06)
Education	Intermediate (Ref)	551 (21)	24.89	1.00 (1.00–1.00)
	Lower	449 (17)	29.75	1.09 (0.94–1.27)
	Higher	410 (15)	18.02	0.98 (0.86–1.12)
	Unknown	1265 (47)	19.20	1.02 (0.92–1.14)
Household and family situation				
Marital status	Unmarried (Ref)	985 (37)	36.80	1.00 (1.00–1.00)
	Married or partnership^b^	1042 (39)	12.87	0.74 (0.64–0.86)
	Divorced	584 (22)	36.26	0.95 (0.84–1.09)
	Widowed	64 (2)	31.20	0.92 (0.71–1.20)
Living situation	Part of couple without child living at home^b^ (Ref)	593 (22)	13.99	1.00 (1.00–1.00)
	Living alone^a^	1219 (46)	51.61	2.37 (2.05–2.74)
	Part of couple with child living at home	659 (25)	12.63	1.02 (0.91–1.16)
	Child Living at Home	51 (2)	28.03	1.37 (1.02–1.84)
	Other Household Member	153 (6)	26.15	1.20 (0.98–1.47)
Socio-economic^1^				
Personal income	1st quartile (Ref)	1112 (42)	37.02	1.00 (1.00–1.00)
	2nd quartile	696 (26)	22.19	0.86 (0.71–1.04)
	3rd quartile	502 (19)	15.95	0.83 (0.68–1.02)
	4th quartile^b^	317 (12)	10.05	0.59 (0.47–0.75)
Household income	1st quartile^a^ (Ref)	1231 (46)	40.79	1.00 (1.00–1.00)
	2nd quartile	602 (23)	19.17	0.79 (0.69–0.91)
	3rd quartile	475 (18)	15.20	0.89 (0.75–1.04)
	4th quartile^b^	319 (12)	10.10	0.76 (0.62–0.93)
Household wealth	1st quartile (Ref)	851 (32)	28.25	1.00 (1.00–1.00)
	2nd quartile	725 (27)	22.98	0.81 (0.71–0.92)
	3rd quartile	548 (20)	17.43	0.90 (0.78–1.03)
	4th quartile	503 (19)	16.07	0.95 (0.82–1.11)
Labour market position & benefits				
	Working^b^	1061 (40)	14.27	0.74 (0.66–0.84)
	Unemployment benefit^2^	243 (9)	30.27	1.18 (0.98–1.42)
	Social assistance^2^	252 (9)	50.65	1.13 (0.96–1.34)
	Disability benefit^a,2^	565 (21)	55.92	1.42 (1.22–1.64)
Health care costs^3^				
Health care costs excluding mental health care	Health care Costs <€5000 (Ref)	2183 (82)	19.15	1.00 (1.00–1.00)
	Health care costs €5000-€10,000^a^	213 (8)	43.05	1.58 (1.34–1.86)
	Health care costs > €10,000^a^	260 (10)	49.62	1.79 (1.53–2.09)
	Health care costs unknown^b^	19(1)	11.31	0.10 (0.06–0.16)
Mental health	Mental health care usage^a^	890 (33)	114.59	5.77 (4.83–6.91)

^a^ indicates ≥40 suicides per 100,000 with significant OR (>1)

^b^indicates <15 suicides per 100,000 with significant OR (<1)

Reference groups are subscripted^a or b^ despite non-significant OR

^1^1^st^ quartile = lowest income or wealth, 4^th^ quartile = highest income or wealth

^2^Reference group represents the opposite category; working vs. not working; unemployment benefit vs. not receiving unemployment benefit; social assistance vs. not receiving social assistance; disability benefit vs. not receiving disability benefit; used mental health care vs. not used mental health care

^3^ Health care (excluding mental health care) is measured as costs per calendar year, mental health as service use per calendar year

In contrast, the suicide rate was lower (< 15 per 100,000) for middle-aged men with a first-generation non-western migration background (9.63 suicides per 100,000), a high personal income (10.05 suicides per 100.000), or high household income (10.10 per 100,000), and those who lived with a partner and child (12.63 per 100,000), were married or had a registered partnership (12.87 per 100,000), lived with a partner without a child living at home (13.99 per 100,000), or were working (14.27 per 100,000). 

#### Combined factors

When analyzing the combinations of factors, it was found that middle-aged men who received mental health care, lived alone, and received disability benefits had the highest number of suicides (289.13 per 100,000), which is approximately 13.6 times higher than the average suicide rate for middle-aged men. Other combinations of factors with a higher suicide rate were: men who received mental health care, lived alone and had a low household income, 9.5 times the average (201.98 suicides per 100,000); men who received mental health care, had a low income, had healthcare costs of <€5000 (excluding mental health care) and didn’t have a migration background, 8.8 times the average (186.83 suicides per 100,000); men who received mental health care, received a disability benefit and were categorized in the 50 to 59 age group, 8.3 times the average (176.56 suicides per 100,000); and men who received mental health care and were categorized in the 60 to 69 age group, 6.9 times the average (147.37 suicides per 100,000). Among combinations where mental health care use was absent, two combinations stood out: income in first quartile, healthcare costs of <€5000 (excluding mental health care), no migration background, age 50–59 (52.27 suicides per 100,000); and income in second quartile and disability benefit (54.73 suicides per 100,000). Other combinations of factors can be found in Table 2.

**Table 2:** Combination of factors outcomes logistic regression model middle-aged (40–70) men from 2013 to 2021

	Factors	Number of suicides (%)	Number of suicides per 100,000 middleaged (40–70) men	Odds Ratio (CI)	Compound Odds Ratio (CI)
Without mental health care	Household wealth in the second quartile + Divorced	133 (5)	30.64	1.08 (0.86–1.35)	0.81 (0.65–1.02)
	Age 40–49 + Unemployment benefits	96 (4)	31.16	1.16 (0.88–1.53)	1.36 (1.10–1.69)
	Income in the first quartile + Household wealth in the first quartile + Living in the Western region	187 (7)	34.48	1.03 (0.83–1.27)	0.66 (0.52–0.83)
	Income in the first quartile + Living alone + Working	145 (5)	36.16	0.80 (0.65–0.98)	1.40 (1.11–1.76)
	Income in the first quartile + Care costs excluding mental health care <€5000 + no migration background	716 (27)	39.92	1.16 (0.95–1.42)	1.16 (0.95–1.42)
	Divorced + Living in the Southern region	160 (6)	40.31	1.02 (0.83–1.26)	0.98 (0.79–1.20)
	Income in the first quartile + Household wealth in the first quartile	461 (17)	41.49	0.77 (0.64–0.94)	0.77 (0.64–0.94)
	Income in the first quartile + Care costs excluding mental health care <€5000 + no migration background + Age 50-59^a^	291 (11)	52.27	1.08 (0.91–1.30)	1.45 (1.16–1.81)
	Income in the second quartile + Disability benefits^a^	208 (8)	54.73	1.19 (0.96–1.47)	1.45 (1.16–1.80)
With mental health care	Mental health care + Married or in partnership	293 (11)	79.58	1.30 (0.99–1.70)	5.59 (4.27–7.32)
	Mental health care + Working + Age 50–59	134 (5)	107.65	1.41 (1.12–1.77)	6.96 (5.40–8.97)
	Mental health care + Married or in partnership + Care costs excluding mental health care <€5000 + no migration background	191 (7)	111.54	1.26 (0.96–1.66)	7.07 (5.68–8.80)
	Mental health care + Lower education^a^	160 (6)	123.95	0.79 (0.63–0.99)	5.01 (3.91–6.40)
	Mental health care + Age 60-69^a^	194 (7)	147.37	1.47 (1.19–1.82)	6.11 (4.84–7.72)
	Mental health care + Disability benefits + Age 50-59^a^	113 (4)	176.56	0.68 (0.53–0.87)	6.37 (4.66–8.72)
	Mental health care + Income in the first quartile + Care costs excluding mental health care <€5000 + no migration background^a^	252 (9)	186.83	0.87 (0.71–1.06)	5.81 (4.47–7.56)
	Mental health care + Household wealth in the first quartile + Living alone^a^	363 (14)	201.98	0.75 (0.61–0.93)	10.26 (8,05–13,07)
	Mental health care + Living alone + Disability benefits^a^	184 (7)	289.13	1.26 (0.99–1.60)	24.29 (17.74–33.25)

^a^ indicates suicide rates ≥ 40 suicides per 100,000 with a significant OR (> 1) for combinations without mental health care, for combinations with mental health care ^a^ indicates ≥ 115 suicides per 100,000 with a significant OR (> 1). 1st quartile = lowest income or wealth, 4th quartile = highest income wealth. Health care (excluding mental health care) is measured as costs per calendar year, mental health as service use per calendar year

### Psychosocial autopsy

Six themes were identified in the psychosocial autopsy data. These themes provide contextualization and extension to the identified risk patterns among middle-aged men and reveal some of the complex mechanisms behind the identified risk factors.

#### Experience of loss

The experience of loss was a major theme in the interviews concerning middle-aged men. Loss events triggered psychological pain, expressed through feelings of sadness, defeat, anhedonia, burdensomeness, and inner restlessness. Proximal losses in the year prior to the suicide stood out, with eleven men experiencing a relationship break-up, while others experienced job loss or the loss of stability (i.e., financial- or housing stability). Notably, these losses typically did not exist in isolation, but interacted and accumulated, contributing to a perceived lack of control over the situation and a sense of displacement and entrapment: *“And look*,* in the last months of his life*,* he had just gotten divorced. (…) He and his second wife had a house in [country]. He had to sell it. He thought it was terrible that he had to sell it. (…) And then he said*,* (…): I feel completely displaced. I don’t have a home anymore; I don’t have a girlfriend anymore. I feel so awful*,* he said.”* (informant 20, brother).

#### Childhood trauma

A second theme was the experience of traumatic events. Eight men experienced traumatic events which frequently had occurred in early life, and these events had an enduring effect on their mental health in midlife. Three men experienced sexual abuse, while the other five men experienced bullying, physical abuse, or witnessing the death of close relatives (including suicides). Feelings of shame and burdensomeness were often attributed to these events. Moreover, men were reluctant to express their feelings and thoughts later in life which respondents linked to these early life events, and often seemed to stem from perceived stigma: *“He always said*,* ‘If I allowed it for so long*,* I must have wanted it.’ It was also by a man*,* about two years*,* it happened between the age of [age omitted; early adolescence]”* (informant 15, daughter) Other experiences in childhood were connected to the families and environments in which the men were raised. Some environments were described as emotionally unsafe, characterized by limited parental affection, neglect, strained parental relationships, and insecure attachment patterns. These circumstances had an enduring impact on the sense of emotional safety and well-being. In the following quote traumatic experiences with someone’s father in combination with emotional suppression was seen as contributing factor to suicide: *“he had a difficult childhood with his father. And I think that already involved something that was never properly processed*,* or never fully explored*,* or something he always tended to keep bottled up inside.”* (informant 29, partner).

#### Disconnectedness

A third emerging theme was a marked sense of disconnectedness within interpersonal relationships, consistent with the interpersonal contributing factors identified in the national data (i.e., living situation and marital status). Disconnectedness was related to various causes, including the experiences of loss or personality traits (e.g., reluctance to share thoughts or emotions). Interpersonal difficulties ranged from tensions with close family, partners, landlords, or colleagues, to experiences of bullying, unmet support needs, or not being able to truly connect with others. In the following quote, the impact of disconnectedness within both the familial and occupational context becomes evident: “*But even before that*,* he was struggling because he had conflicts with people at practically every job. That weighed heavily on him. He had a very bad relationship with our daughter. She barely came to see him anymore. And yes*,* those were major factors*,* and ultimately*,* they were the final straw.”* (informant 12, ex-partner) As a result, many men experienced feelings of loneliness or isolated themselves, leading for some to a lack of fulfillment within their interpersonal needs. Interpersonal disconnectedness was strongly observed among eleven men who lived alone and perceived as instrumental to the suicide of at least seven men. By contrast, this influence was observed in only a few men who did not live alone. Importantly, the sense of disconnectedness was not limited to the immediate interpersonal environment but was also experienced in a wider social context, especially for five men deviating from heteronormative norms. Their disconnectedness was driven by a subjective experience of rejection and isolation, and even (threats of) physical violence. The following quote illustrates how society did not embrace the decedents’ identity, and how this made it a struggle to belong. “*That really bothered him. […] It was very clear to him that he was indeed gay. And he embraced it. But at the same time*,* he struggled with it. That was because others didn’t embrace it.”* (informant 23, brother).

#### Focus on stabilization as opposed to improvement

Eighteen men received some form of mental health care at the time of their suicide. They either were in contact with a medical professional, were undergoing treatment, or were on a waiting list for specialized mental health care. The majority of respondents expressed their dissatisfaction with care, ranging from failure to adequately involve close ones, inadequate care, poor therapeutic engagement, and insufficient interventions. Most commonly, men in treatment had persistent or episodically recurring mood disorders. The long-term use of psychotropics, specifically antidepressants and antipsychotic medication was often reported. Respondents expressed their frustration about how healthcare professionals seemed to focus on stabilization through medication without trying to achieve actual improvements. *“He just received a new drug*,* and so he called the psychiatrist again*,* and he said: “my head is going crazy from those pills. So*,* she said: ‘well*,* so then just quit again [name]. We will try something else.’ And now he had something that seemed to work*,* but not sufficiently*,* clearly. […] I thought it such a shame that the psychiatrists -they have all these questionnaires and so on- that they did not try to look at: what is underlying all this? What is behind this? I am truly sorry about that. And then just: a pill*,* and another pill.”* (Informant 22, partner).

In nine cases, adverse side effects were reported prior to their death, mostly involving anti-depressants. Notably, five men were in a titration phase, which is known to affect depressive and suicidal feelings. Some proxy-informants indicated that the adverse side effects of medication in this phase contributed to suicide, as shown in this quote: *“Well*,* we also think he was truly overcome by that*,* or rather*,* truly overcome by*,* but then again*,* that peak of medication side effects. Because the police were able to backtrack a bit to when he died*,* somewhere between [time] and [time]*,* I believe. That’s roughly the middle of the day when those side effects are at their peak.”* (informant 2, brother).

#### Physical problems, pain and debilitation

In thirteen cases, the physical health of the deceased was described as poor, often linked to a sense of suffering and a decline in mental well-being. Eight men experienced physical pain, including hip and back problems (e.g., herniated discs), headaches, or pain elsewhere in the body. In some cases, these symptoms added to an already existing mental burden and were perceived by the respondents as contributing factor to suicide. Five of these men received benefits (i.e., disability benefit, retirement) and were not working. In the following quote, it becomes clear how pain could make someone feel isolated and debilitated. *“In the years we were together*,* he had so*,* so*,* so much pain*,* he couldn’t do anything. But truly*,* nothing. He used medications*,* heavy painkillers that could tranquilize a horse. I can imagine that if you are always in pain*,* that he thought: okay*,* this [bedridden] on top. I can’t leave my bed. I have no one.”* (informant, 30, ex-partner) Associated with this debilitation was an impact on autonomy. This was emphasized for a man who lost his sight due to trauma, which was perceived a key factor for his suicide: “*Yeah*,* I don’t think he could have accepted that. Just the whole combination*,* so to speak. I think if he had—if he hadn’t gone blind completely*,* yeah*,* he would have been able to function reasonably well again*,* because he hadn’t really suffered that much damage*,* so yeah*,* because of his blindness [decisive factor for suicide].”* (informant 18, daughter) Notably, in a least seven men, signs of functional debilitation was observed prior to their suicide. This debilitation was characterized by eating problems, weight loss, sleeping difficulties, lack of energy, and alcohol use. Hence, men were described as having become a mere shadow of who they once were. The following quote illustrates the physical and behavioral consequences of mental decline: *“You saw it right away [dysphoria] — he was losing kilos*,* and of course*,* he started drinking and smoking*,* he didn’t sleep anymore*,* he just became very skinny. And from*,* well*,* a prosperous Dutch man*,* it was immediately noticeable with him. You could actually see it on him. A bit of a sunken face*,* he became very thin.”* (informant 29, partner).

#### Maladaptive coping through alcohol use

Proxy informants indicated that nine men had long-term problems with alcohol consumption. Alcohol use was often portrayed as a coping mechanism and could be traced back to their upbringing, where drinking was normalized as strategy for coping with emotions. Excessive alcohol use could lead to problems at work, friction in social and romantic relationships, and a worsened physical wellbeing. The following quote illustrates the consequences of long-term alcohol use on interpersonal relationships and how it tapped into experiences of loss: “*I think that if you’re at the beginning or mid-career*,* you can be a high-functioning alcoholic. But at some point it stops. And if that starts to literally erode your brain capacity and your morals and values*,* then I don’t think there’s much left for you to truly see the light of day.”* (informant 10, daughter) For three men, alcohol use happened secretly, deliberately hiding it from their partner. Nevertheless, partners would notice shifts in behavior, such as aggression, or discover evidence like receipts from liquor stores. Crucially, although alcohol problems were indicated for nine men, only two men were diagnosed with an addiction. Proxy informants revealed that the deceased often did not perceive their alcohol use as problematic and therefore did not experience the need to seek professional support.

## Discussion

### Risk factors

The aim of this mixed-methods study is to deepen our understanding about contributing factors to suicide among middle-aged men, using quantitative analyses to identify risk patterns and qualitative data to contextualize them. Between 2012 and 2021 the average suicide rate of middle-aged men was 21.25 per 100,000. Nationwide data indicated associations of (mental) health problems, socioeconomic difficulties, and social isolation with suicide. Moreover, it suggests a cumulative impact of having multiple factors, whereby for example, experiencing psychological problems, receiving a disability benefit, and living alone, resulted in a suicide rate 13.6 times higher than the average. The psychosocial autopsy data contributed by contextualizing mental health problems and revealed interconnections between physical, social, and socioeconomic factors. Moreover, it directly linked psychosocial risk factors to suicide and uncovered insights that could not be identified in the nationwide data such as experiences of loss, childhood trauma, disconnectedness, debilitation, stabilization-oriented system of mental health care, and maladaptive coping through alcohol use.

First, the findings reveal the prominent role and influence of health-related factors. Having mental health problems stood out as a key characteristic both individually and in interaction with other factors. Physical health issues also affected suicide risk, as indicated by health care utilization and further contextualized by qualitative insights about disabilities and functional debilitation. The impact of psychological and physical problems on midlife suicide risk was also shown in a review by Qin [10], who found that mood disorders (primarily depression) and musculoskeletal disease had a strong link to suicide. Our qualitative data suggest that proximal factors (e.g., recent losses and stressors, and medication side-effects), more distal factors (e.g., prolonged mental health struggles, alcohol use, and trauma), or a combination of both greatly influenced the psychological well-being of middle-aged men. Furthermore, the data suggests that a proportion of men were in contact with mental health services in the year prior to their death. This points to possible gaps in adequacy of care and services, which are also described by proxy-informants who critique the current biomedical narrow focus. This is supported by Deacon [35], who states that the biomedical model systematically neglects psychological treatment processes and inhibits psychosocial interventions, thereby reducing attention to the underlying drivers of distress. Perhaps, a more holistic care approach that integrates attention to physical complaints, psychological distress, and social factors alongside pharmaceutical interventions would better serve men who experience complex health issues.

Second, socioeconomic disadvantages, such as unemployment, low income, financial problems or accommodation problems played an important role in the suicide of middle-aged men, reinforcing associations with suicide demonstrated in earlier research [9, 10]. These adversities disproportionately affect men compared to women [36], which may be influenced by societal norms regarding the role of being a provider and successful role model [15] that make economic stability a central part of a men’s identity [37]. Consistent with previous findings, our study showed that having a disability benefit - which often signals long-term functional impairment, social marginalization (Chen et al., [38] ;Gedikli et al., [39]; Klein et al., [40]), and financial strain [41] – was frequently observed among men who died by suicide. Moreover, similar to research by Choi [42], the dominance of socioeconomic disadvantages became even more evident in interaction between variables. This was also prominent in the qualitative data, where recent socioeconomic losses showed a profound impact on overall well-being of men. Consequently, this indicates that less “well-off” middle-aged men are particularly vulnerable to adverse life events, which subsequently amplifies the suicide risk profile. Hence, it illustrates the interconnectedness of these disadvantages and the need for multileveled prevention strategies.

Third, sociodemographic findings situated at the interpersonal level, such as living alone and being unmarried, were highly present among men who died by suicide. These factors were further contextualized by an experience of disconnectedness. Living alone is associated with increased levels of psychiatric morbidity [43, 44], subjective loneliness and lack of perceived emotional support [45, 46], influencing suicide risk. During midlife, living alone may be a result of separation, divorce, or widowhood, often leading to social isolation and psychological distress [47]. Interview data further revealed the impact of recent separation, which is identified as a critical period [48, 49]. Moreover, midlife is characterized by shifts in family structures, career transitions, and changes in health status, all of which can further strain interpersonal relationships and needs [6]. This effect may be particularly pronounced for men who live alone. Taken together, these findings reveal the pivotal role of interpersonal connectedness, and the potential consequences of its absence.

The findings underscore that suicide risk emerges from the interplay between clinical factors and broader social and contextual factors, aligning with the findings among middle-aged men in the UK [12]. The impact of the interacting factors in many cases vastly exceeds the sum of the individual factors. Therefore, they shine light on the importance of considering factors beyond mental ill-health [50]. When clinical vulnerability intersects with socioeconomic challenges and isolation, the ability to stay resilient seems to weaken drastically. Furthermore, there are indications that cultural norms surrounding masculinity have an impact on the overall risk, e.g., by leading to difficulties in emotional expression or to feelings of failing to meet the norms when experiencing loss [15]. Targeting only one factor, like improving access to services, fails to capture the complexity of risk among middle-aged men. Hence, as emphasized in existing literature [16, 51, 52], comprehensive, multileveled prevention strategies that simultaneously ensure continuous care in both clinical and community contexts, address socioeconomic instability, and strengthen social support networks are needed.

### Protective factors

Although this study was designed to identify risk factors, several protective factors surfaced from the national dataset. Data indicated that middle-aged men with a first-generation non-Western migration background were less likely to die by suicide than men with no migration background (9.63 vs. 22.5 suicides per 100,000). Empirical findings show mixed and heterogeneous tendencies [53–55]. Notably, there is a lack of research specifically addressing middle-aged men with a first-generation migration background. Lower suicide rates observed in this study may be attributed to cultural and religious beliefs about suicide [56], community cohesion, positive ethnic group identity, communalism [57], or health selection processes among refugees (i.e., where individuals who migrate may be healthier than those wo do not [58]). However, these findings should be interpreted with caution, as men with a migration background are highly heterogenous, and generalization may not be appropriate.

Other protective factors identified correspond to the inverse of the risk factors found. For instance, economic prosperity, such as high (household) income, serves as a protective factor. Greater financial resources may diminish exposure to the psychosocial stressors that trigger psychopathology and suicidal ideation [59]. The same applies for being in a relationship. This is consistent with other studies where interpersonal connections [60] and marriage [61] serve as protective factors. Finally, having a job is indicated as a protective factor. Having employment not only offers financial stability but also forms a foundational element of social identity and offers a framework of structure and routine [59]. Thus, maintaining financial stability, meaningful relationships, and employment all appear to play a pivotal role in mitigating mental health problems and suicide risk.

### Strengths and limitations

What distinguishes this study is its comprehensive examination of cumulative sociodemographic effects on suicide within a large dataset, further enriched by in-depth interviews. By analyzing ten years of suicide data, this study uncovers in-depth insights that establish a robust foundation for risk factor assessment. However, this study also has limitations. Within the national dataset the sociodemographic factors were limited to the available factors, possibly missing important factors (e.g., type of mental health care). Also, mental health problems were measured based on the use of services, potentially overlooking men who experienced problems but did not seek help [62]. Although this may not capture all men, service utilization provides an objective source of data and is the most accessible method in this context. Whilst the nationwide dataset reveals associations and patterns relevant to suicide prevention, it cannot establish causality. The psychological and intentional processes leading to suicide remain unclear from registry data alone, and the risk profiles identified should therefore be interpreted as probabilistic patterns. Furthermore, regarding the psychological autopsy, twelve interviews are often sufficient to reach saturation in qualitative interviews [63], but it is unclear whether more are needed for a phenomenon as complex as suicide. This may also relate to the interview design, which did not explicitly elicit cultural aspects, despite their potential importance [15]. It is also possible that within subpopulations such as those with a migrant background or non-heterosexuality, additional factors would emerge that were not captured in this study. Additionally, the interpretation of the proxy-informants should be considered, as it may differ from those of the deceased, especially since some men were described as emotionally closed off and may not have fully articulated their thoughts and feelings [64]. To mitigate this bias, two informants were included where possible. It should also be considered that for men who are more socially isolated a proxy informant may not have been available. Yet, psychological autopsy remains one of the most informative approaches for understanding the complexity of risk factors and offers possibilities to make a direct link to death by suicide [24].

### Implications

#### Practice

Due to the cumulative impact of clinical, contextual, and sociodemographic factors we recommend adopting interventions both on a systematic and individual level. This encompasses structural measures such as expanding social and economic support, and individual interventions aimed at strengthening coping mechanisms and managing other stressors (Choi et al., 2022). This multidisciplinary approach to health and social care seems necessary to signal, refer and intervene with middle-aged men. First, awareness needs to be raised about suicide risk factors among stakeholders across these different domains. Subsequently, further research is needed into the roles of relevant stakeholders and the resources they can offer to realize effective coordination.

A particular focus should be placed on middle-aged men who experience (mental) health challenges, encounter economic adversity, are socially isolated, or have work related problems. Our findings indicate that many men are in contact with health services before their suicide, emphasizing the crucial role of these services in suicide prevention. For the clinical setting it requires equipping primary care providers with screening capabilities and means to intervene with men [65, 66]. This could entail direct suicide risk assessment [50] while also fostering trust and respect within the health professional and patient relationship [67]. Additionally, a more socially oriented approach that captures the interdisciplinary nature of intervening should be implemented. This entails placing a focus on experiences of loss, as bereaved accounts frequently linked events such as relationship breakups and job loss to the deceased’s sense of hopelessness and feeling unable to cope [68]. Moreover, in established disability support systems like the Netherlands, multiple opportunities exist where middle-aged men at risk can be identified and targeted for interventions (e.g., when work absence begins, during income support applications, throughout workplace rehabilitation, and during benefit assessments [69]).

Furthermore, this study underscores the need to focus on social connection. Empirical data from the UK indicates that community-based support services can be important for middle-aged men to connect with others [70]. It seems crucial that men experience a sense of contribution and shared purpose, since being positioned as someone in need of help can push them away. This approach may be particularly relevant for men who live alone, have gone through a relationship breakup, or experience interpersonal disconnection.

#### Research

Realizing suicide prevention interventions and adequate policies for middle-aged men requires research from various domains. First, it is important to assess the support needs and help-seeking behaviors of men with lived experience. This allows for the tailoring of interventions to the specific needs of middle-aged men. Second, to realize a multidisciplinary coordinated approach, it is crucial to focus on analyzing service utilization of middle-aged men across various domains, to identify patterns, gaps and opportunities. Third, given the significant cumulative effect of factors, research needs to pinpoint more risk patterns that can be the primary focus of preventive interventions. This includes factors beyond the scope of this research, such as the relationship between clinical, genetic, familial, and neurobiological factors. Furthermore, the influence of cultural masculinity norms on suicide risk appears notable and calls for a deeper investigation. These principles also hold for protective factors, as their inclusion in prevention efforts has been shown to improve well-being and quality of life, preventing suicidal processes from developing in the first place [71].

## Conclusions

This mixed methods study allowed for both the identification of risk patterns of middle-aged men who died by suicide and a deeper understanding of psychosocial characteristics. The findings suggested that the cumulative effect of (mental) health, socioeconomic, and interpersonal hardships carry significant risk for middle-aged men. The multifactorial nature of suicide among middle-aged men highlights the complexity of its development and gives direction for preventive actions. Moreover, interventions must be designed, implemented, and maintained within the system where middle-aged men are currently situated, encompassing clinical settings, community contexts, and broader social support structures. Future research should focus on the needs of middle-aged men and the complex network of stakeholders, examining their potential roles in suicide prevention, with the ultimate goal of providing tailored care and support.

## Supplementary Information

Additional file 1.

Additional file 2.

Additional file 3.

## Data Availability

The datasets analyzed in this study are not publicly available due to ethical restrictions. Statistics Netherlands data is highly secured and can only be accessed through secure servers, sharing is not permitted. The interview dataset contains sensitive information that may allow the identification of participants: the Medical Research Ethics Committee (MREC) of Amsterdam University Medical Centre has imposed this restriction (registration number: 2023.0246). Contact: metc@amsterdamumc.nl, Medisch-ethische toetsingscommissie | Amsterdam UMC. Data may be obtained from the author (JM) upon reasonable request.

## Abbreviations

CBS: Central Bureau of Statistics Netherlands
COR: Compound odd ratios
COREQ: Consolidated criteria for reporting qualitative research
ISCED: International Standard Classification of Education
MREC: Medical Research Ethics Committee of Amsterdam University Medical Center
OR: Odds ratios
SD: Standard deviation
