# Supplementary material for: Insights into middle-aged men (40–70) who died by suicide: a nationwide mixed methods study in the Netherlands
Source: BMC Public Health. 2026 Jun 2;26:2432. doi: 10.1186/s12889-026-27854-8 (PMC13501754; doi:10.1186/s12889-026-27854-8)
Supplement: Supplementary file 2 [file 12889_2026_27854_MOESM1_ESM.docx]

**Additional file 1**

Characteristics of participants

Table A. Characteristics of proxy informants and cases of middle-aged men who died by suicide

|  | **Respondents (n = 30)** |
| --- | --- |
| **Proxy informant, n (%)** |  |
| (ex)partner | 16 (53.3%) |
| peer / sibling | 6 (20%) |
| child | 8 (26.6%) |
|  | **Cases (n = 25)** |
| **Age, mean (SD)** | 54.6 (7.7) |
| **Psychological disorder** *^a^***, n (%)** | 13 (52%) |
| **Migration background, n (%)** |  |
| no migration background | 23 (92%) |
| **Education (ISCE***^b^***), n (%)** |  |
| lower (0-2) | 6 (24%) |
| middle (3-4) | 5 (20%) |
| higher (5-8) | 14 (56%) |
| **Marital status, n (%)** |  |
| divorced | 10 (40%) |
| married | 10 (40%) |
| Unmarried | 5 (20%) |
| **Sexuality, n (%)** |  |
| heterosexual | 20 (80%) |
| homo/bi/other sexual | 5 (20%) |

*Note. ^a^* Including affective disorders (i.e., depression and bipolar disorder), personality disorders, neurodevelopmental disorders, substance-related and addictive disorders, and anxiety disorders. *^b^* International Standard Classification of Education: 0: Early childhood education (‘less than primary’ for educational attainment);1: Primary education; 2: Lower secondary education; 3: Upper secondary education; 4: Post-secondary non-tertiary education; 5: Short-cycle tertiary education; 6: Bachelor’s or equivalent level; 7: Master’s or equivalent level; 8: Doctoral or equivalent level.
