# Supplementary material for: Insights into middle-aged men (40–70) who died by suicide: a nationwide mixed methods study in the Netherlands
Source: BMC Public Health. 2026 Jun 2;26:2432. doi: 10.1186/s12889-026-27854-8 (PMC13501754; doi:10.1186/s12889-026-27854-8)
Supplement: Supplementary file 3 [file 12889_2026_27854_MOESM2_ESM.docx]

**Additional file 2**

Interview instrument

Interview instrument psychosocial autopsy middle aged men (40-70). This instrument was developed by the research team leading the longitudinal psychosocial autopsy study at 113 Suicide Prevention (The Dutch national center for suicide prevention and crisis support).

***Reconstructive Interview***

- Create calm circumstances when conducting the interview, such as in a quiet room, preferably in someone’s home.
- Provide an explanation of the topics that will be covered during the interview.
- Offer a time estimate and include breaks between different sections.
- Not all questions apply to every case and may be skipped as necessary.
- Rephrase each question in your own words when possible, but strictly adhere to the content. Adapt the questions to the gender and the relationship of the interviewee with the deceased (e.g., your daughter/son, name, etc.).
- Some questions in the instrument include examples for clarification (e.g., about social media). Use these only if the respondent seems to misunderstand the question.
- For narratives: delve deeper rather than broadening, and only follow up on the provided story. This means follow-up questions should aim to clarify what has already been said, understand its role in the suicide, and avoid introducing new themes. This minimizes speculation by the bereaved and helps keep the interview duration manageable.

**0. Introduction**

**0.1** We always find it helpful to have a sense of the person we’re discussing. Do you have a photo of [name] that we may see?
 **0.2** What kind of person was [name]?
 **0.3** What did [name] enjoy doing in their free time?

Engage in a conversation with the bereaved about what you see in the photo. Take a moment to observe the space and discuss any objects (e.g., an altar) that refer to the deceased.

**1. Narrative – Key Contributing Factors**

We’d like to begin with your impression of what led to [name]’s suicide. Looking back on [name]’s life, what do you believe contributed to their death by suicide?

- Follow-up questions should delve deeper, not broaden the scope, focusing on clarifying what has been said and understanding how it played a role in the suicide. Avoid introducing new themes to reduce speculation and manage interview duration.

**1.1** Was there a moment when the problems could have been noticed? By whom could these problems have been observed?
 **1.2** What were the first signs of these problems, and when did they begin?
 **1.3** Are there things or issues you wish had gone differently or been handled differently?

**2. Reconstruction of the Last Months**

**2.1 Narrative Account of the Last Months**

**2.1.1** We now ask you to focus on the last months of [name]’s life. What can you tell us about these final months leading up to the suicide?
 Details can be valuable to us, and you are the person who experienced [name] in their final period. Share everything you remember and noticed.

**2.1.2** What was the primary daily routine of [name] (e.g., work, hobbies, social contacts)?

- **If employed in the period before the suicide:**
  - Reiterate questionnaire answer (e.g., Question G8): Is it correct that [name] worked as a …?
  - What was their mood like at work in the final months?
  - How were their workplace relationships in the final months?
  - Did they attend work in this period? Was there frequent absenteeism?
  - Were there workplace factors you associate with the suicide (e.g., team changes, bullying, performance pressure, possible dismissal)?
- **If retired in the period before the suicide:**
  - Do you think retirement played a role for [name]?
  - Did you notice a change in their sense of purpose? Did they lose a significant purpose with retirement? Did a new purpose arise, or did they miss one?

**2.2 Contact in the Last Months and Final Contact**

**2.2.1** How was your contact with [name] in their final months?
 **2.2.2** How often did you interact with them during this period?
 **2.2.3** What was their mood like in the final months?

- Did [name] express hopelessness?
- Could they still view the future positively?
- Did they ever feel like a burden to others?
- Did they feel like they didn’t belong anywhere?
- Were there changes in their mood or behavior you associate with their death?

**2.2.4** When was the last time you had contact with [name]?
 **2.2.5** How did they behave during this final contact? What was their appearance like?

**2.3 Suicide-Related Communication**

**2.3.1** Looking back, do you think [name] had suicidal thoughts in their final months?
 **2.3.2** Did they say or write anything suggesting they were preparing for or thinking about suicide (e.g., social media posts)?
 **2.3.3** Did you notice any behaviors indicating they were preparing for suicide (e.g., online activity, such as deleting accounts or buying gifts for loved ones)?

- **If they never expressed suicidal feelings:**
  - Why do you think [name] refrained from talking about their thoughts or feelings?

**2.4 Adverse Life Events in the Last Months**

**2.4.1** Are there significant events in [name]’s final months you associate with the suicide? What happened, and how did they react?

**2.5 Preparation**

**2.5.1** Looking back, do you think [name] planned or prepared for their suicide?
 **2.5.2** What gives you that impression (e.g., researching, scouting locations, setting a date, saying goodbye, ordering means)?

**2.5.3** Were substances (e.g., alcohol, medications) involved?

**2.5.4** Was [name] alone at the time of death? If not, who was present?

**2.6 Farewell Message**

**2.6.1** Was a note, message, or object left behind?

- Who was it addressed to, and did it state reasons for the suicide?
- Can we see or photograph the message/object, with your permission?

**2.7 Method and Circumstances of Death**

These questions concern the method and circumstances, which may be painful. Please feel free to take a break if needed.

**2.7.1** Who discovered [name] after their death?
 **2.7.2** Where did the death occur? What is the deceased's relationship with this location?
 **2.7.3** How did they acquire the means used in their suicide?
 **2.7.4** Why do you think they chose this specific method?

**2.8 Closing**

Is there anything else you think is important to share about [name]’s suicide that we haven’t discussed?
 Would you suggest speaking with someone from their workplace, such as a colleague or employer, for the second interview?
